# Social and economic costs and health-related quality of life in stroke survivors in the Canary Islands, Spain

**DOI:** 10.1186/1472-6963-12-315

**Published:** 2012-09-12

**Authors:** Julio Lopez-Bastida, Juan Oliva Moreno, Melany Worbes Cerezo, Lilisbeth Perestelo Perez, Pedro Serrano-Aguilar, Fernando Montón-Álvarez

**Affiliations:** 1 University Castilla-La Mancha, Talavera de la Reina, Toledo, Spain; 2 Evaluation Unit, Canary Islands Health Service, Canary Island, Spain; 3 CIBER de Epidemiología y Salud Pública (CIBERESP), Barcelona, Spain; 4 Red Temática de Investigación en Envejecimiento y Fragilidad (RETICEF), Barcelona, Spain; 5 Nuestra Señora de la Candelaria University Hospital, Canary Islands Health Service, Canary Island, Spain

**Keywords:** Stroke, Cost-of-illness, Health care costs, Informal care, Loss of labor productivity, Health-related quality of life, Spain

## Abstract

**Background:**

Cost-of-illness analysis is the main method of providing an overall vision of the economic impact of a disease. Such studies have been used to set priorities for healthcare policies and inform resource allocation. The aim of this study was to determine the economic burden and health-related quality of life (HRQOL) in the first, second and third years after surviving a stroke in the Canary Islands, Spain.

**Methods:**

Cross-sectional, retrospective study of 448 patients with stroke based on ICD 9 discharge codes, who received outpatient care at five hospitals. The study was approved by the Research Ethics Committee of Nuestra Señora de la Candelaria University Hospital. Data on demographic characteristics, health resource utilization, informal care, labor productivity losses and HRQOL were collected from the hospital admissions databases and questionnaires completed by stroke patients or their caregivers. Labor productivity losses were calculated from physical units and converted into monetary units with a human capital-based method. HRQOL was measured with the EuroQol EQ-5D questionnaire. Healthcare costs, productivity losses and informal care costs were analyzed with log-normal, probit and ordered probit multivariate models.

**Results:**

The average cost for each stroke survivor was €17 618 in the first, €14 453 in the second and €12 924 in the third year after the stroke; the reference year for unit prices was 2004. The largest expenditures in the first year were informal care and hospitalizations; in the second and third years the main costs were for informal care, productivity losses and medication. Mean EQ-5D index scores for stroke survivors were 0.50 for the first, 0.47 for the second and 0.46 for the third year, and mean EQ-5D visual analog scale scores were 56, 52 and 55, respectively.

**Conclusions:**

The main strengths of this study lie in our bottom-up-approach to costing, and in the evaluation of stroke survivors from a broad perspective (societal costs) in the first, second and third years after surviving the stroke. This type of analysis is rare in the Spanish context. We conclude that stroke incurs considerable societal costs among survivors to three years and there is substantial deterioration in HRQOL.

## Background

Stroke is the second leading cause of death after ischemic heart disease (IHD), and the third leading cause of disability-adjusted life years in high-income countries after IHD and unipolar depressive disorders [1]. Up to 16% of patients die within the first month after their stroke [2,3], and within 1 year after the stroke event nearly 55% die or experience severe consequences [4]. About one half of survivors are left with permanent disabilities and have significant needs for rehabilitation and long-term care [5].

Apart from large costs for home- and hospital-based rehabilitation and care, there are also other societal costs related to stroke, such as direct non-healthcare costs (formal and informal care) and productivity losses. Aging of the population may further increase demands on the healthcare system due to stroke in the future [6], which makes stroke-related costs an important area of study.

Previous studies have assessed the impact of stroke on health-related quality of life (HRQOL) with the EuroQol EQ-5D questionnaire [7-13]. The main findings suggest that stroke survivors have significantly impaired HRQOL, and that the burden of nonfatal stroke should thus be recognized more widely. Measures of HRQOL yield information that is not provided by traditional outcome scores; accordingly, some studies support guidelines to measure HRQOL in stroke research [8].

Traditionally, health has been evaluated objectively based on observation or medical interventions, and taking into account general indicators such as life expectancy, mortality and disease prevalence. However, these indicators have lost part of their predictive value in wealthy societies where diseases tend to be chronic, mortality rates are extremely low and life expectancies have reached new heights. This scenario calls for concepts and measures of health that are more dynamic, and a strictly biomedical model is being replaced by one that includes patients’ assessments of their own heath [14,15].

The Canary Islands Health Service considers that information on HRQOL, together with other information sources such as epidemiological and socioeconomic information, plays a key role in priority-setting and resource allocation among different health problems in healthcare planning [16]. In recent decades HRQOL has increased in importance as a key health status indicator.

In the Canary Islands, with a population close to 2 million, the demographic structure, aging rate, health expectations and infant mortality are similar to the mean values for all of Spain [17]. The National Health System in each of Spain’s 17 regions guarantees free access and universal coverage, as well as an identical healthcare package for every citizen [18]. Furthermore, ratios of healthcare resources (professionals, structural and technological equipment) in the Canary Islands are in line with average values in Spain [19]. Therefore, there is no reason to assume that the cost of resources in our study differs substantially from elsewhere in Spain. This study was designed to examine stroke costs using different approaches. First, we analyzed the societal costs incurred by patients during their first, second and third year after surviving a stroke event. Second, we analyzed their HRQOL in the first, second and third years after the stroke. Finally, we identified associations between HRQOL and societal costs.

## Methods

### Research design and subjects

This was a cross-sectional, retrospective study of people diagnosed with stroke who received outpatient care and were living in the community. Patients were recruited from five hospitals in the Canary Islands, Spain. All patients with stroke were admitted to the hospital. They were not initially seen by a general practitioner.

The patients were divided into three categories: 1-, 2- and 3-year survivors after stroke (group 1, group 2 and group 3, respectively). All patients and caregivers were informed about the study objectives and data confidentiality, and were asked to indicate their understanding of the study conditions and agreement to participate by signing an informed consent form. The study was approved by the Research Ethics Committee of Nuestra Señora de la Candelaria University Hospital.

### Information and variables of interest

The fieldwork was carried out between January and December 2004. The questionnaires were administered by postal survey. The information sources used in the study were the hospital admissions database and a self-completed questionnaire completed by patients or their caregivers [20-22]. Demographic and clinical data were collected from patients diagnosed previously with stroke and caregivers.

To estimate resource utilization, the questionnaire solicited information covering the 6-month period prior to the study (12 months for hospital admissions). Data for the preceding 6 months were extrapolated to the entire year. We considered 6 months to be an appropriate recall period [21,22]. Information on hospital admissions was obtained from hospital databases. Patients or their caregivers were asked about reductions in working time and any temporary and permanent disability, and these data were used to calculate losses of labor productivity. Information about HRQOL was collected from survivors with the generic EQ-5D questionnaire [23].

### Costing methodology

We used the prevalence approach to estimate costs. Disease prevalence takes into account all existing cases during a given year and all resources used for prevention, treatment and rehabilitation, plus losses as a result of morbidity and mortality within that year. Prevalence-based cost-of-illness analysis has the advantage of incorporating measurements of total annual healthcare expenditure, which is particularly relevant for chronic conditions such as stroke that require long-term treatment [20-22]. In this context, a bottom-up costing approach was used to estimate total and average annual costs [24].

Data on resources were collected for each patient. The resources used were multiplied by unit costs to estimate the annual cost per patient, with 2004 as the reference year. A societal perspective was also used in the study. Two types of direct costs were considered: those derived from healthcare and non-healthcare costs (formal and informal care). Labor productivity losses were estimated from physical units converted into monetary units with an approach based on human capital theory [25].

### Direct healthcare costs

Direct costs were derived from healthcare utilization. The value of resources used by patients was calculated in terms of the relevant unit costs and the average cost per patient in the sample. Information about the number of hospital admissions was obtained from the admissions databases of each of five hospitals in the Canary Islands Health Service. Inpatient care was based on diagnosis-related group (DRG) costs. Unit costs were estimated with a microcosting method (not charges for reimbursement) for DRGs (unit costs published by the Spanish Ministry of Health) based on the average cost per DRG from 18 hospitals in Spain (including one hospital from the Canary Islands) [26].

Data for the volume of outpatient care (rehabilitation, medical tests and examinations, visits to health professionals and home medical care) and the number of emergency visits were obtained from the questionnaires. The unit cost was obtained from the SOIKOS healthcare cost database [27], then multiplied by the resource quantities. SOIKOS is the most complete database used in Spain to obtain healthcare unit costs. The sources of unit costs were published articles, reports, hospital accounting systems, etc.; the figures are updated every year.

Information regarding the medications consumed by patients with stroke was obtained from the questionnaires. The cost of drugs used by patients was calculated by determining the daily cost for each of the products used (based on the cost of each package dispensed and the dose used), and was then multiplied by duration of use. When no information concerning the number of units per package was available, we assumed the largest dispensation package for drugs. The costs of prescription drugs used were obtained from the list of approved drugs in Spain [28].

Information concerning the use of orthopedic devices and healthcare-related transportation was obtained from the questionnaires. The costs of orthopedic devices were obtained from the SOIKOS healthcare unit cost database [27], distribution firms, the Canary Islands regional legislative record and the Spanish parliamentary record.

### Direct non-healthcare costs

Informal care is defined as the performance of tasks by nonprofessionals that help maintain or enhance patient independence. Therefore, informal services are defined as the group of tasks or care provided by nonprofessional caregivers, who are often relatives but may also be friends or neighbors. Information about informal care was obtained from the questionnaires, specifically from the items concerning the time spent helping the patient with his or her basic daily activities, and the time spent helping with necessary instrumental daily activities.

To estimate time used to provide care to patients, we used the opportunity cost of the time spent by the informal caregiver, which in economic terms is equal to the wage the caregiver would have earned if an alternative paid activity had been performed [29]. For the main and secondary caregiver, this method assigns a cost per hour based on the gross wage for a domestic cleaner.

Information on formal care provided by social services and professional caregivers was obtained from the questionnaires.

### Loss of labor productivity

Data on loss of labor productivity were obtained from physical units converted into monetary units with a human capital-based approach [25]. Gross wages before the deduction of taxes and social security contributions are considered a good proxy for labor productivity losses. According to human capital theory [25], the average earnings (wage) of a worker are considered a reasonable measure of labor productivity, and can be used as the basis for estimating future wages that go unearned if a worker leaves the labor market as a result of an illness or accident. Labor productivity is described in terms of a worker’s remuneration in the labor market. Thus, our calculations were based on average gross wage figures in the 2004 Wage Structure Survey of the Spanish National Statistics Institute [30]. Annual losses of labor productivity were estimated for the year 2004.

### Patient outcomes

The EQ-5D is a simple generic instrument developed by a multidisciplinary group of researchers [23]. This questionnaire has been validated in Spain and is commonly used in economic evaluation and technology assessment. It has also been used for periodic health surveys in two regions of Spain – Catalonia and the Canary Islands – where the populations are similar [31].

There are five dimensions in the EQ-5D covering the areas of mobility, self care, everyday activities, pain/discomfort and anxiety/depression. A total of 243 possible health states can be defined in this way. Evaluations of these health states have been reported for the general population [32]. The values or utilities are indicated on a scale on which 0 is the value of death and 1 is the value of perfect health. For statistical analysis, the scores on the EQ-5D were divided into four categories of HRQOL: very poor (EQ-5D index scores less than 0), poor (EQ-5D index scores between 0 and 0.5), fair (EQ-5D index scores between 0.51 and 0.85) and good (EQ-5D index scores higher than 0.85). The main reason is that although the set of social values in the EQ-5D is apparently a continuous variable, gaps or discontinuities in the distribution of scores may make it more informative to interpret the findings as a categorical variable for the purposes of fitting statistical models and interpreting the results.

### Statistical models

Because of the different distributions of the main items, different estimation methods were chosen. Healthcare costs were analyzed with a log-normal multivariate model. Productivity losses were analyzed with a probit model, a type of discrete response model in which the variable of interest is a binary variable that takes a value of 0 if there is no productivity loss, and a value of 1 if there is productivity loss. Because of the high percentage of the sample who were past the legal retirement age (65 years), the analysis was done initially with the whole sample, and then only with people under 65 years of age. To analyze informal care costs, an ordinate probit model was used in which the dependent variable takes a value of 0 if the person received no informal care, a value of 1 if the person received less than 60 hours per week, and a value of 2 if more than 60 hours of informal care were received per week.

The aim of the models was to determine whether HRQOL was significantly related to the number of years elapsed since the stroke, with age and sex as control variables. To this end we used an ordinary least squares model (robust for heteroscedasticity) for the effects of healthcare costs, a probit model to identify the likelihood of job losses, and an ordered probit model to analyze the effects of the amount of informal care received.

## Results

A total of 480 questionnaires were collected (34% of all questionnaires sent) from people with stroke, 32 of which were excluded because the information they contained was insufficient or inadequate. There was no difference in sex or age distribution between patients who responded to the questionnaire and those who did not.

The main characteristics of our sample are shown in Table 1. The estimated average cost per person was €17 618 after 1 year of survival, €14 453 after 2 years and €12 924 after 3 years (Table 2). Informal care costs made up the largest proportion, at 60% of the total average per person cost after 1 year of survival, 77% after 2 years and 76% after 3 years. Healthcare expenditures ranged between 28% of the total expenditure for 1-year survivors and 15% for 2-year and 3- year survivors. The cost of productivity losses was less than informal care and total healthcare costs. This is not surprising in light of the high average age of the participants, most of whom had left the labor market at the moment of their stroke. Two thirds (65%) of the participants were more than 65 years old, the legal retirement age in Spain.

**Table 1:** Characteristics of the sample and population

**Interview sample**	**Total (n=448)**	**Group 1 (n=94)**	**Group 2 (n=205)**	**Group 3 (n=149)**
**Average age (years)**	67.1 (12.2)	67.9 (11.8)	67.3 (12.3)	66.4 (12.1)
**Sex**				
Male	56.7%	55.3%	57.6%	56.4%
Female	43.3%	44.7%	42.4%	43.6%
**Is there a main caregiver?**				
Yes	62.9%	64.9%	66.3%	57.1%
No	37.1%	35.1%	33.7%	42.9%
**Is there a second caregiver?**				
Yes	32.59%	28.7%	38.1%	27.5%
No	67.41%	71.3%	61.9%	72.5%
**Informal care hours per week (whole sample)**	35.0 (35.3)	34.8 (34.6)	36.9 (34.7)	32.5 (36.6)
**Informal care hours per week at least one caregiver)**	55.1 (29.2)	52.8 (29.5)	54.9 (28.4)	57.0 (30.7)
**Health-related quality of life (EQ-5D VAS)**	53.69 (26.29)	55.96 (26.62)	51.64 (27.04)	55.03 (24.98)
**Health-related quality of life (EQ-5D index score)**	0.4708 (0.4388)	0.4961 (0.4246)	0.4674 (0.4407)	0.4596 (0.4475)

Expressed as mean or percentage (Standard deviation in parentheses).

**Table 2:** Annual average costs of stroke survivors after 1, 2 and 3 years in Canary Islands, Spain

	**1year (n=94)**	**2years (n=205)**	**3years (n=149)**
**Direct healthcare costs (in €)**			
Acute hospitalization	2,827 (879)	63 (660)	14 (171)
Rehabilitation	462 (903)	472 (935)	274 (748)
Drugs	752 (660)	635 (649)	773 (813)
Tests	283 (535)	510 (1 576)	332 (540)
Emergency department care (acute)	91 (261)	90 (187)	93 (380)
Outpatient and primary healthcare visits	149 (301)	99 (179)	152 (230)
Medical home care	17 (67)	18 (65)	13 (45)
Orthopedic devices	105 (179)	99 (178)	77 (156)
Healthcare transportation	321 (816)	185 (478)	164 (453)
**Subtotal**	**5007 (2 238)**	**2173 (2 430)**	**1894 (1 652)**
**Direct non-healthcare costs (€)**			
Caregiver’s time costs (informal care)	10 523 (10 440)	11 158 (10 495)	9828 (11 050)
Social services	161 (612)	127 (677)	228 (853)
**Subtotal**	**10 684 (10 575)**	**11 285 (10 572)**	**10 057 (11 336)**
**Total, Direct costs**	**15 691 (11 464)**	**13 457 (11 374)**	**11 951 (11 879)**
**Loss of labor productivity (in €)**			
**Subtotal**	**1926 (4616)**	**995 (3396)**	**973 (3522)**
**Total costs**	**17 618 (12 633)**	**14 453 (11 914)**	**12 924 (12 314)**

Expressed as mean (Standard deviation in parentheses).

With regard to the HRQOL of people who have survived a stroke, the EQ-5D index score was 0.47 over 1, and the EQ-5D VAS score was 53.69 over 100 (Table 1). These scores are significantly lower than in the general population, after controlling for age and for other chronic diseases [33]. Also of note is that the HRQOL was similar in 1-, 2- and 3-year survivors. This indicates that after a stroke, some survivors will recover but others will suffer permanent sequelae, which means that personal autonomy will be limited (dependence) and their HRQOL will remain low.

Table 3 shows the results of the statistic analyses of costs related to the main explanatory variables. Time since the stroke event and HRQOL were explanatory variables, whereas age, age squared and sex were used as control variables. Informal care, healthcare costs and productivity losses were analyzed separately because of differences in their characteristics and distribution. Formal care at home (part of non-healthcare direct costs) was not included because it accounted for only a small proportion of the overall cost.

**Table 3:** Statistical analysis of healthcare costs, productivity losses and informal care costs

	**Log healthcare costs**	**Loss of labor productivity, probit model marginal effects**		**Informal costs, ordered probit model marginal effects**			
	**Total**	**Total**	**<65years old**	**Total**	**High costs (>60hours per week)**	**Moderate costs (1–60hours per week)**	**Without informal care**
Age	0.055 ** (0.026)	0.0298** (0.011)	0.177** (0.077)		0.0105 (0.012)	0.0003 (0.0007)	−0.011 (0.012)
Age-squared	−0.0005** (0.0002)	−0.0003** (0.0001)	−0.0017 **(0.0007)		−0.0001 (0.0001)	−0.00002 (0.000001)	0.0001 (0.0001)
Female	−0.131 (0.104)	−0.062** (0.029)	−0.309 ** (0.0703)		0.028 (0.040)	0.0007 (0.0017)	−0.029 (0.0412)
Patient Group 2^¥^	−1.306** (0.095)	−0.088** (0.035)	−0.223 ** (0.087)		−0.0002 (0.0515)	−0.00001 (0.0017)	0.0002 (0.0531)
Patient Group 3^¥¥^	−1.446** (0.114)	−0.085** (0.0314)	−0.237 **(0.082)		−0.0402 (0.0545)	−0.0021 (0.0044)	0.0422 (0.0566)
HRQOL Fair	0.436** (0.146)	(0.020) (0.043)	0.0095 (0.103)		0.181** (0.0535)	−0.004 (0.010)	−0.176** (0.050)
HRQOL Poor	0.875** (0.165)	0.131** (0.069)	0.343** (0.126)		0.275** (0.068)	−0.0467* (0.0257)	−0.229** (0.048)
HRQOL Very poor	1.201** (0.151)	0.282** (0.081)	0.319** (0.149)		0.526** (0.058)	−0.145** (0.041)	−0.382** (0.039)
Sample	424	424	154	423	129	155	139
F (8,415)	38.88	78.02	40.97	76.48			
R-squared	0.3179	0.2090	0.2135	0.0823			

Reference category: men, patient group 1, HRQOL good.

Standard deviation between brackets.

** Statistically significant at 95% confidence level.

* Statistically significant at 90% confidence level.

^¥^ 2-year survivors.

^¥¥^ 3-year survivors.

The variables age, time of the stroke event and HRQOL significantly affected healthcare costs. The age–cost curve was concave, and was greatest at age 55 years. Sex did not affect healthcare costs, whereas the effect of stroke event was significant. The reason is that healthcare costs during the first year are strongly conditioned by the initial hospitalization. In marginal terms and after controlling for the other variables, health expenditures were on average €2900 less for 2-year survivors, and €3200 less for 3-year survivors, with differences in hospital expenditures accounting for most of the decrease with time. The influence of HRQOL on costs was reflected in our findings: a poor HRQOL was significantly associated with greater healthcare expenditure. For example, expenditures for patients with an HRQOL score less than 0 (very poor HRQOL) were more than €2000 greater than in patients with a better HRQOL.

Age, sex, time of stroke event and HRQOL were significantly related to the likelihood of positive productivity losses. When men under 65 years of age were used as the reference group, women were 31% less likely than men to experience productivity losses. However, this result should be regarded with caution because the proportion of women in the Spanish labor market is much lower than the proportion of men. Therefore, the likelihood of temporary or permanent loss from the labor market is expected to be larger in men, since this group made up the larger proportion of wage-earners before the stroke event. Age, sex and years of survival were not significantly related to the quantity of informal care received. In contrast, HRQOL was a significant variable. The lower the quality of life, the greater the likelihood of receiving more than 60 hours of informal care. Compared to participants with a good HRQOL, people with a very poor, poor or fair HRQOL were 53%, 28% and 18% more likely, respectively, to have received more than 60 hours/week informal care. The same reasoning was used to analyze the marginal effects of HRQOL without informal care. People in the very poor HRQOL category were 38% less likely to receive informal care than a person with good HRQOL.

## Discussion

In recent decades, stroke has been a major health-related problem with important social consequences in high-income countries. The high incidence and prevalence of stroke and its social consequences in terms of mortality, morbidity and economic costs justify the attention this diagnosis has received from health authorities and society in general.

A variety of international studies have analyzed stroke-related costs [34-45]. There is general agreement that total direct healthcare expenditure on stroke in high-income countries accounts for 3% to 6% of all national health service expenditure [36,37,39-42]. During the first year after the stroke event, in-patient expenditure represents about 75% of total healthcare costs [45,46]. However, in comparison to other neurodegenerative diseases such as Alzheimer’s disease, the inclusion of societal costs is a recent phenomenon in cost-of-illness studies of stroke. Nevertheless, informal care for stroke patients may be a significant hidden cost to society [47-49].

The present analysis highlights the importance of studying the economic consequences of stroke and interpreting the results in an international context. Although the ideal design for bottom-up studies of the societal costs of stroke is a population-based cohort study, the results of our analysis provide insights into the distribution of the costs of stroke and the impact of stroke on national expenditures for healthcare.

We show that in 2004, the estimated annual cost per stroke survivor was €17 618 for the first year, €14 453 for the second year and €12 924 for the third year. By analyzing healthcare and non-healthcare costs separately, we found that informal care costs increased in relative terms during the years following the stroke. The composition of healthcare costs evolved during the 3 years after the stroke. During the first year, informal care and hospitalization costs were the largest expenditures. However, for 2-year and 3-year survivors, the largest costs were for informal care, productivity losses, drugs and rehabilitation.

The high cost of informal care in this study may have several possible explanations. First, the methodology we used may have influenced our estimates. In earlier studies, indirect costs included costs related to both job loss and informal care. Cost-of-illness studies published more recently, however, use more precise classifications of the items that contribute to societal costs. Second, because of our study design, people in institutions were excluded from the analysis. Moreover, in Spain, home-based social services are limited compared to other countries in central and northern Europe [50-52]. Therefore, the main caregivers for disabled people are their family members; consequently informal care costs are very high.

The present study quantifies the activity of informal caregivers, and also highlights the importance of informal care. Informal stroke care represents a significant hidden cost to Spanish society. Because the Spanish population is aging rapidly, the formal and informal care burden may increase significantly in the future. The present study also shows that non-medical home care (private transport and social services) represents only 5% of direct non-healthcare costs. However, social changes (incorporation of women into the labor market, smaller family size, greater social demand for professional care) point to a growth in the need for formal care in the coming years.

Productivity losses were greatest in first-year survivors, a finding that may reflect the return to the labor market of second- and third-year survivors.

HRQOL is another source of information that helps to define the global impact on society of a specific health problem; is also useful to set priorities and allocate resources, together with other information sources such as incidence, prevalence, mortality and costs. Knowledge of HRQOL is also needed to measure the effectiveness of health interventions on disease management. Despite the relative frequency of stroke, this diagnosis is characterized by a substantial economic and HRQOL burden. Stroke survivors with poor or very poor HRQOL are more likely to have positive productivity losses compared to people with fair and good HRQOL. In the sample we studied, poor or very poor HRQOL was related with a 30% greater likelihood of productivity losses.

Multivariate analysis showed that the average cost per patient was significantly lower in 2- and 3-year survivors compared to 1-year survivors. The high cost of hospital services during the first year of survival probably accounts for this finding.

### Limitations

Further, our estimates for survivors of stroke may be overestimated since there was no adjustment for pre-stroke costs and co-morbidity. This may be reflected more strongly in the 2nd and 3rd year costs [53].

Our choice of variables and the econometric model were restricted by the explanatory variables available in the database. Age and sex were the only socioeconomic variables included in the patient survey. Another limitation of our study was that we had no information regarding clinical evidence of event severity. We would expect that more severe events in survivors would be predictive of higher costs. HRQOL and time elapsed since the stroke (1, 2 or 3 years) were used as proxy variables to measure the degree of severity. These variables explained not only direct costs but also informal care costs. Although our choice of survival times may have been too broad, the use of a greater number of time intervals is not recommended since this would impose limits on the sample size.

Several other limitations deserve mention. The most important is the use of cross-sectional data from a study done in the Canary Islands (Spain), which may limit the generalizability of our conclusions. However, the five participating hospitals in the Canary Islands were selected to reflect potential differences in diagnostic and therapeutic patterns. Furthermore, the ratios of professional, structural and technological healthcare resources in the Canary Islands are in line with average values in Spain [19]. Therefore, there is no reason to assume that the cost of the resources consumed by the regional centers we studied differs substantially from costs elsewhere in Spain.

Another major limitation of this study is the fact that the costs of institutionalization and long-term care were not included. Although nursing home availability in Spain is increasing, actual use remains quite low, forcing most potential residents to receive home care. Another limitation is potential recall bias, given that patient-based data were obtained by questionnaire.

The retrospective analysis used in the present study entails a risk of bias in sample selection. The findings may thus have been influenced by our lack of information about the characteristics of patients who died during the second and third year after their stroke.

Despite the limitations of cost-of-illness analysis studies, the governments of many countries and regions continue to encourage researchers to carry them out. The reason is that decision-makers consider information about the financial impact of diseases to be a useful input for program planning [54]. This information does not replace, but rather complements, epidemiological information on population-level health problems.

The Spanish Ministry of Health and Social Policy has included estimates of the associated costs of stroke in a recent policy document titled *Strategies for Stroke in the National Health System*, in order to document the social significance of this health problem with a view to proposing measures for stroke prevention and care [55]. This growing level of interest suggests that the ability of cost-of-illness analysis to help us understand the social impact of diseases may allow this approach to become a useful tool for designing public policies [55,56].

In spite of these limitations, we believe that this study represents the most complete and realistic costing to date of the burden of stroke in the Canary Islands and Spain. The main strength of the study lies in the bottom-up approach to costing. In addition, the costs were estimated for a period of 3 years rather than 1 year, thus our estimated costs provide a more accurate picture of the medium-term burden of stroke.

Two previous studies have analyzed healthcare costs in terms of labor productivity losses and informal care costs related to stroke in Spain [57,58]. One study has examined the costs of formal and informal care (non-healthcare costs) together with HRQOL [59]. However, ours is the first study to consider societal costs and HRQOL associated with surviving a stroke event in Spain. We found that most of the societal cost is represented by informal care. Additionally, we show that worse HRQOL is associated with greater social costs of stroke.

A clear understanding is needed of the current patterns of resource use, costs, and HRQOL in stroke survivors in order to inform health services planning appropriately. Cost-of-illness studies need to be updated to understand the economics of diseases and their changing cost structures. This will enable policymakers to achieve a better understanding of the factors that have an impact on stroke-related expenditures, and will also enable a better-informed distribution of resources.

## Conclusions

Stroke incurs considerable societal costs even in survivors, including very high economic costs and a deterioration in health-related quality of life. Informal stroke care represents a significant hidden cost to Spanish society. Because the Spanish population is aging rapidly, the burden of formal and informal care may increase significantly in the future.

## Abbreviations

IHD: Ischemic heart disease; HRQOL: Health-related quality of life; DRG: Diagnosis related group; EQ-5D: EuroQol EQ-5D.

## Competing interests

The authors declare that they have no competing interests.

## Authors’ contributions

JLP and JOM conceived and designed the research, acquired the data, analyzed and interpreted the data, performed the statistical analyses, handled funding and supervision, drafted the manuscript and critically revised the manuscript for important intellectual content. MWC acquired the data, analyzed and interpreted the data, performed statistical analyses and critically revised the manuscript for important intellectual content. LPP conceived and designed the research, analyzed and interpreted the data, performed statistical analyses and drafted the manuscript. PSA conceived and designed the research, acquired the data, analyzed and interpreted the data, drafted the manuscript and critically revised the manuscript for important intellectual content. FMA conceived and designed the research, acquired the data, analyzed and interpreted the data and critically revised the manuscript for important intellectual content. All authors read and approved the final manuscript.

## Sources of funding

This study was supported by grant number 031263 from the Fondo de Investigaciones Sanitarias (FIS) and by Merck, Sharp and Dohme de España, S.A.

## Pre-publication history

The pre-publication history for this paper can be accessed here:

http://www.biomedcentral.com/1472-6963/12/315/prepub
